# Minimal Residual Disease as a Predictive Factor for Relapse after Allogeneic Hematopoietic Stem Cell Transplant in Adult Patients with Acute Myeloid Leukemia in First and Second Complete Remission

**DOI:** 10.3390/cancers4020601

**Published:** 2012-06-20

**Authors:** Rada M. Grubovikj, Asif Alavi, Ahrin Koppel, Mary Territo, Gary J. Schiller

**Affiliations:** 1 Hematological Malignancy, Stem Cell Transplant Program, David Geffen School of Medicine at UCLA, 10833 Le Conte Avenue, Room 42-121 CHS, Los Angeles, CA 90095, USA; E-Mail: gschiller@mednet.ucla.edu; 2 Department of Medicine, David Geffen School of Medicine at UCLA, 10833 Le Conte Avenue, Room 42-121 CHS, Los Angeles, CA 90095, USA; E-Mails: asifalavi@gmail.com (A.A.); mterrito@mednet.ucla.edu (M.T.); 3 Tahoe Forest Cancer Center, Division of Hematology and Oncology, Department of Internal Medicine, University of California, Truckee, CA 96160, USA; E-Mail: ahrinbk@gmail.com

**Keywords:** minimal residual disease, allogeneic hematopoietic stem cell transplantation, acute myeloid leukemia

## Abstract

Allogeneic hematopoietic stem cell transplantation (allo-SCT) is potentially curative for patients with high-risk leukemia, but disease recurrence remains the leading cause of treatment failure. Our objective was to determine the impact of minimal residual disease (MRD) by any technique in adult patients with acute myeloid leukemia (AML) in morphologic first and second complete remission undergoing allo-SCT. Fifty nine patients were eligible for the study of 160 patients transplanted over ten years. For the MRD assessment we used multiparametric flow cytometry, cytogenetics and fluorescent *in situ* hybridization; 19 patients (32.2%) were identified as MRD positive. Patients with MRD had a consistently worse outcome over those without MRD, with 3-years leukemia-free survival (LFS) of 15.8% *vs*. 62.4% and overall survival (OS) of 17.5% *vs*. 62.3%. Relapse rate was significantly higher in MRD-positive patients; 3 years relapse rate in MRD-positive patients was 57.9% *vs*. 15.1% in MRD-negative patients. Detection of MRD in complete remission was associated with increased overall mortality (HR = 3.3; 95% CI: 1.45–7.57; *p* = 0.0044) and relapse (HR = 5.26; 95% CI: 2.0–14.0; *p* = 0.001), even after controlling for other risk factors. Our study showed that for patients in morphologic complete remission the presence of MRD predicts for significantly increased risk of relapse and reduced LFS and OS.

## 1. Introduction

Allogeneic hematopoietic stem cell transplantation (allo-SCT) is potentially curative for patients with high-risk leukemia, but disease recurrence remains the leading cause of treatment failure [1,2]. Many patients relapse due to persistence of low numbers of residual leukemia cells that are undetectable by conventional cytomorphologic criteria. Patients with fewer than 5% blasts in the bone marrow are generally considered to be in complete clinical remission, based on morphologic disease characteristics [3], and are eligible for allogeneic transplant, yet the burden of leukemia in defining a morphologic threshold of 5% may still be great [4]. Achieving clinical remission, as defined by traditional methods, is insufficient to predict for a long-term remission. The persistence of blasts below the threshold of morphologic detection, identified by sensitive molecular and immunologic tests, is termed minimal residual disease (MRD). Improvements in disease detection may allow for opportunities to intervene in order to prevent relapse, or equally, consider restricting access to transplant. Over the last three decades, advances in deciphering the cytogenetic and molecular abnormalities underlying the pathogenesis of acute myeloid leukemia (AML) provide a potential means to identify those at risk for relapse after allo-SCT [5,6,7]. Current methods to detect MRD include cytogenetics, fluorescence *in situ* hybridization (FISH), flow cytometry (FC) and qualitative and quantitative real-time polymerase chain reaction (RT-PCR) [8]. Most sensitive for follow-up monitoring is the PCR methodology which achieves sensitivities of 10^−4^ to 10^−6^, detecting 1 abnormal cell in 10,000 to 100,000 normal cells. However, nearly 40% of patients with AML have no cytogenetic or molecular markers suitable for PCR monitoring [9]. Laboratory data suggest that AML originates from a rare population of cells, termed leukemia stem cells (LSCs) or leukemia-initiating cells. At least some of these cells persist after treatment and are probably responsible for disease relapse. Still, the similarities and differences between LSCs and residual leukemia cells detected after treatment in MRD assays are not fully understood. Although, unfortunately, in AML no leukemia-specific antigens are detectable immunophenotypically, leukemia blasts often display aberrant or uncommon phenotypes that may also be used as markers of MRD [10]. Immunophenotyping by flow cytometry provides an excellent option for monitoring of MRD, targeting patients across all subgroups [11]. Aberrant cells are detectable by multicolor FC with sensitivities ranging from 10^−2^ to 10^−4^, detecting 1 abnormal cell in 100 to 10,000 normal cells. Compared to conventional cytogenetic analysis, FISH allows for delineation of specific numerical and structural chromosome aberrations in interphase cells (interphase cytogenetics). FISH allows a quantification of cells carrying the aberration and the detection of chromosome abnormalities for which no PCR assays are available [12]. Very few studies of MRD in AML patients in morphologic remission undergoing allogeneic transplant have been reported, and the clinical significance of MRD detection by a variety of laboratory tools is still not clear. The objective of this study was to determine the impact of minimal residual disease, identified by any technique, in adult patients with AML in morphologic first and second complete remission undergoing allo-SCT, and to determine whether the MRD as defined by these techniques, would be predictive for adverse outcome.

## 2. Experimental Section

Our study group consisted of AML patients in first or subsequent complete remission treated in the Hematologic Malignancies/Stem Cell Transplant Unit at the UCLA, from January 2000 through January 2010 using allogeneic bone marrow, blood-derived and cord blood stem cells from histocompatibile related and unrelated donors. Cytogenetic risk groups at diagnosis were classified as follows: favorable—t(8;21)(q22;q22), t(15;17)(q22,q21), inv(16)(p13q22)/t(16,16)(p13;q22); intermediate—entities not classified as favorable or unfavorable; and unfavorable risk—abn(3q)[excluding t(3;5)(q21~25;q31~35)], inv(3)(q21q26)/t(3,3)(q21;q26), add(5q), del(5q), −5, −7, add(7q)/del(7q), t(6;11)(q27;q23), t(10;11)(p11~13;q23), other t(11q23)[excluding t(9;11)(p21–22;q23) and t(11;19)(q23;p13)], t(9;22)(q34;q11), −17/abn(17p), complex (≥4 unrelated abnormalities) [13]. Patients were divided in two groups according to their remission status (first or second complete remission). Assessment was done according to their disease status prior to transplantation (first or second remission), *de novovs*. secondary AML (sAML), initial cytogenetics, type of transplantation (related [R] *vs*. unrelated [U]), source of transplantation (bone marrow [BM], peripheral blood stem cell [PBSC], cord blood [CB]), HLA matching, conditioning intensity (myeloablative *vs*. reduced intensity) and conditioning protocol (total body radiation [TBI] *vs*. non-TBI). Secondary AML was defined as leukemia arising from preleukemia or after cytotoxic treatment for another malignancy or clonal neoplasm [14]. Tissue typing for HLA-A, B, C, and DRB1 was by high resolution for BM and PBSC donors, and for HLA-A, B (by low resolution) and DRB1 (by high resolution) for CB donors. Donor-recipient pairs were classified as matched (8/8 for BM and PBSC and 6/6 for CB) or mismatched (7/8 for BM and PBSC, 5/6 and 4/6 for CB) [15]. Patients were followed after transplantation for relapse, treatment related mortality (TRM), leukemia-free survival (LFS) and overall survival (OS). All patients were treated on institutional review board-approved protocols and gave consent in accordance with the declaration of Helsinki. Follow up was current as of February 2011.

### 2.1. MRD Assessment

Each patient evaluable for this study underwent bone marrow (BM) biopsy prior to transplant, and the BM samples were further investigated for persistence of MRD. All BM aspirates and biopsy samples were analyzed at the UCLA Department of Pathology and Laboratory Medicine. For the MRD assessment we used multiparameter flow cytometry, cytogenetics and fluorescent *in situ* hybridization (FISH). Cytogenetics and FISH analyses were performed according to the standard protocols. In flow cytometry, residual tumor cells were detected using immunofluorescence of surface markers. A panel of at least three antibodies selected on the basis of the immunophenotype of the original leukemia was used. MRD was identified as a cell population showing deviation from normal antigen expression patterns compared with normal or regenerating marrow. Any level of residual disease was considered MRD positive. Patients who had tissue involvement (leukemia cutis) with negative bone marrow were also considered for MRD assessment (patient #52).

### 2.2. Statistical Analyses

OS was calculated from the date of transplant until death from any cause, and surviving patients were censored at last follow-up. TRM was defined as death due to causes unrelated to underlying disease. LFS was calculated from the date of transplant until death or relapse, and patients who were alive and disease-free were censored at last follow up. Patient survival curves were estimated using the Kaplan Meier (KM) method and compared across groups using the log rank test. Cumulative incidence curves for relapse were estimated while adjusting for the competing risk of mortality. Cumulative incidence curves for TRM were estimated while adjusting for the competing risk of relapse or non-transplant related mortality. Incidence curves were compared across groups using the Fine and Gray test.

Hazard ratios (HRs) for mortality, relapse, LFS and TRM were computed under the Cox model. For relapse and TRM the Cox model was expanded to a competing risk Cox model to allow for competing risk. Risk factors were compared between the MRD-positive *vs*. MRD-negative groups to determine whether they were comparable. Comparisons were carried out using the exact chi-square test (categorical variables), *t*-test (continuous variables) or Wilcoxon rank sum test for trend (ordinal variables). To evaluate the relationship between MRD and all-cause mortality after controlling for potential confounders we used the multivariate Cox regression model. No multivariate analysis was carried out for relapse or TRM due to the limited number of events. Risk factors were initially screened using univariate Cox regression. The initial multivariate model included the following five factors that were found to be significant or marginally significant (*i.e*., *p* < 0.25) based on the initial screen: initial cytogenetic, previous disease/prior SCT, age at transplant, transplant type/source and conditioning intensity. To select the final model we used the backwards stepwise procedure with *p* < 0.25 as the retention criterion. All analyses were done with SAS version 9.2 (Copyright © 2002–2008 by SAS institute Inc., Cary, NC, USA). A *p* value of ≤0.05 was considered significant in all cases.

## 3. Results

One hundred and sixty (160) AML patients underwent allogeneic stem cell transplantation, but only 59 patients fulfilled criteria to be included in the study on the basis of the: age 18–65, complete morphologic remission, and an available and evaluable bone marrow biopsy done at least 60 days prior to the transplantation with no intervening treatment, or skin biopsy where applicable (patient #52). The remaining 101 patients were not eligible for the study on the basis of age outside the study criteria (18 patients), disease status (48 patients), lack of bone marrow biopsy prior to transplantation in our database or biopsy done more than two months prior to transplantation (30 patients) and five patients who received non-myeloablative therapy. Characteristics of the study group, including transplantation characteristics and conditioning regimens used in the study are shown in Table 1.

**Table 1:** Characteristics of the study group (2000–2010).

Characteristics		Value				
		CR1	CR2			Total (n/%)
		MRD+ (n/%)	MRD− (n/%)	MRD+ (n/%)	MRD− (n/%)	
**Patients (number, %)**		10 (100%)	19 (100%)	9 (100%)	21 (100%)	59 (100%)
**Sex**						
	Male	4 (40%)	6 (31.6%)	5 (55.5%)	13 (90.5%)	28 (47.5%)
	Female	6 (60%)	13 (68.4%)	4 (44.4%)	8 (38.1%)	31 (52.5%)
**Age**						
	Range	28–62	20–61	29–65	30–65	20–65
	Median	51	44	48	43	48
	Mean	51.0	42.4	49.0	46.3	46.3
**Initial cytogenetics**						
	Favorable	/	/	/	1 (4.8%)	1 (1.7%)
	Intermediate	4 (40%)	7 (36.8%)	5 (55.5%)	17 (81%)	33 (55.9%)
	Unfavorable	6 (60%)	10 (52.6%)	2 (22.2%)	1 (4.8%)	19 (32.2%)
	Unknown	/	2 (10.5%)	2 (22.2%)	2 (9.5%)	6 (10.2%)
**Diagnosis**						
	AML *de novo*	4 (40%)	13 (68.4%)	7 (77.7%)	17 (81%)	41 (69.5%)
	Secondary AML	6 (60%)	6 (31.6%)	2 (22.2%)	4 (19%)	18 (30.5%)
**Previous Stem Cell Transplant**						8 (13.6%)
	Autologous	/	/	3 (33.3%)	4 (19%)	7 (11.9%)
	Allogeneic	/	/	/	1 (4.8%)	1 (1.7%)
**Disease status at transplantation**						
	CR1	10 (100%)	19 (100%)	/	/	29 (49.1%)
	CR2	/	/	9 (100%)	21 (100%)	30 (50.8%)
**ECOG status**						
	0	3 (30%)	10 (52.6%)	1 (11.1%)	4 (19%)	18 (30.5%)
	1	1 (10%)	2 (10.5%)	3 (33.3%)	3 (14.3%)	9 (15.2%)
	2	1 (10%)	/	1 (11.1%)	3 (14.3%)	5 (%)
	3–4	/	1 (5.3%)	1 (11.1%)	/	2 (%)
	unknown	5 (50%)	6 (31.6%)	3 (33.3%)	11 (52.4%)	25 (%)
**MRD defined by**						
	Flow cytometry	3 (30%)	/	6 (66.6%)	/	9 (15.2%)
	Cytogenetics	4 (40%)	/	4 (44.4%)	/	8 (13.6%)
	FISH	5 (50%)	/	2 (22.2%)	/	7 (11.9%)
	Leukemia cutis	/	/	1 (11.1%)	/	1 (1.7%)
**Type of transplant**						
	Related	6 (60%)	10 (52.6%)	6 (66.6%)	12 (57.1%)	34 (57.6%)
	Unrelated	4 (40%)	9 (47.4%)	3 (33.3%)	9 (42.9%)	25 (42.4%)
**Graft type**						
	PBSC	6 (60%)	11 (57.9%)	4 (44.4%)	16 (76.2%)	37 (62.7%)
	Bone marrow	1 (20%)	3 (15.8%)	3 (33.3%)	3 (14.3%)	10 (16.9%)
	Cord blood	3 (30%)	5 (26.3%)	2 (22.2%)	2 (9.5%)	12 (20.3%)
**Donor-recipient HLA match**						
	Matched (8/8, 6/6)	7 (70%)	14 (73.7%)	7 (77.7%)	18 (85.7%)	46 (78%)
	Mismatched (7/8, 5/6)	2 (20%)	1 (5.3%)	2 (22.2%)	2 (9.5%)	7 (11.9%)
	Mismatched (4/6)	1 (10%)	4 (21%)	/	1 (4.8%)	6 (10.2%)
**Condition intensity**						
	Myeloablative	9 (90%)	19 (100%)	7 (77.7%)	17 (81%)	52 (88.2%)
	Reduced intensity	1 (10%)	/	2 (22.2%)	4 (19%)	7 (11.9%)
**Conditioning protocol**						
	Non TBI	5 (50%)	11 (57.9%)	4 (44.4%)	12 (57.2%)	32 (54.3%)
	TBI	5 (50%)	8 (42.1%)	5 (55.5%)	9 (42.9%)	27 (45.7%)
**Relapse**						
	Yes	5 (50%)	2 (10.5%)	6 (66.6%)	4 (19%)	17 (28.8%)
	No	5 (50%)	17 (89.5%)	3 (33.3%)	17 (81%)	42 (71.2%)
**Present status**						
	Alive	2 (20%)	12 (63.2%)	2 (22.2%)	11 (52.4%)	27 (45.7%)
	Diseased	8 (80%)	7 (36.8%)	7 (77.7%)	10 (47.6%)	32 (54.3%)

MRD: minimal residual disease; MRD+: patients with MRD; MRD−: patients without MRD; CR1: first complete remission; CR2: second complete remission; M: male; F: female; AML: acute myeloid leukemia; ECOG: Eastern Cooperative Oncology Group; FISH: fluorescence *in situ* hybridization; PBSC: peripheral blood stem cell; TBI: total body irradiation.

There were 19 MRD-positive patients (32.2%) identified with any of the previously mentioned methods, 10 patients in CR1 and nine patients in CR2. Patients defined by FC alone, cytogenetics+FISH, cytogenetics+FC and leukemia cutis had worse outcome than those defined by cytogenetic or FISH alone. There were no survivors in groups when two modalities identified MRD, and only one patient in the FC group survived. In the FISH-positive group both patients are alive, and in the cytogenetics group one patient died and one survived. There was no statistically significant difference in the MRD technique used in predicting the adverse outcome. The main cause of death in MRD-positive patients was relapse, seen in 11 patients (57.9%), four patients died of TRM (21%) and four survived (21%). Detailed characteristics of the MRD-positive patients are shown in Table 2.

**Table 2:** Characteristics of MRD-positive patients.

Pt.#	Age	Cytogenetics (initial)	Dg.	Prior SCT	Rem. status	Flow cytometry	Cytogenetics	FISH	Type of transplant	Conditioning protocol/intensity	Relapse	aGVHD	cGVHD	TRM	Cause of death	OS
10	61	Normal	AML	No	CR1	2–3% myeloblasts	**ck**	/	R 8/8 PBSC	NonTBI/Reduced intensity	+85	0	Yes	No	L, G, OF	+138
11	62	46, xy, del(7)(p15)[23]/47,xy,+8[7]	sAML	No	CR1	1% coexpressing CD34+/CD117+mb	**47, xy,+8**	**Abn. signal pattern, +8**	R 8/8 PBSC	TBI/Myeloablative	+102	4	No	No	L	+196
12	45	normal	AML	No	CR2	**3.5% abn mb**	46, xx	Normal signal pattern	R 8/8 PBSC	TBI/Myeloablative	+210	1	Yes	No	L, G	+299
14	48	normal; FISHdel16p13	AML	Auto SCT	CR2	4% myeloblasts	**46, xy, del16 (q23,q23)**	**Abn. signal pattern, del 16p**	R 8/8 PBSC	NonTBI/Myeloablative	+88	4	No	No	L, G	+96
21	43	normal	AML	No	CR2	**2% mb, 25–30% monocytes**	46, xy	/	R 8/8 PBSC	NonTBI/Reduced intensity	+245	1	No	No	L	+370
22	50	normal	AML	No	CR1	**15–20% monocytes**	46, xy	/	R 8/8 PBSC	TBI/Myeloablative	No	1	No	+82	I	+82
25	49	normal	AML	Auto SCT	CR2	<2% myeloblasts	**ck**	/	U 6/6 CB	NonTBI/Myeloablative	No	0	No	Alive	Alive	>+1512
28	46	t(11q23, 17q25)/t(11,17)	sAML	No	CR1	1% myeloblasts	46, xx	**MLL gene rearrangement 11q23**	R 8/8 BM	TBI/Myeloablative	+260	3	No	Alive	Alive	>+370
31	29	unknown	AML	No	CR2	**1% abn mb**	46, xy	/	R 8/8 PBSC	NonTBI/Myeloablative	+42	0	No	No	L	+98
32	56	ck	sAML	No	CR2	/	**ck**	**Abn. signal pattern, +9/del7q**	R 8/8 BM	NonTBI/Reduced intensity	+110	1	No	No	L	+126
35	61	unknown	AML	Auto SCT	CR2	**3% abn monocytic cells**	46, xx	/	U 7/8 BM	NonTBI/Reduced intensity	+179	2	No	No	L	+184
39	45	normal	AML	No	CR2	**5% abn mb 10% CD34+/CD117+mb**	46, xx	/	U 5/6 CB	TBI/Myeloablative	No	3	No	Alive	Alive	>+1051
43	48	ck	sAML	No	CR1	1% mb	**ck**	**Abn. signal pattern 5q−, 7q−**	U 4/6 CB	NonTBI/Myeloablative	+601	1	No	No	L	+662
46	28	del7q−	AML	No	CR1	**10% CD34+/CD117+mb**	46, xx	Normal signal pattern	U 5/6CB	NonTBI/Reduced intensity	No	0	No	No	L	+269
50	41	del5q,7q	sAML	No	CR1	**4% abn.mb**	/	/	U 8/8 PBSC	TBI/Myeloablative	+114	2	No	No	L	+156
52	61	normal	sAML	No	CR1	**Leukemia cutis**	46, xy	/	R 8/8 PBSC	TBI/Myeloablative	No	4	No	+86	H, G	+86
53	52	47, xx,+8	AML	No	CR1	<2% mb	46, xx	**Abn. signal pattern, +8q**	U 5/6 CB	TBI/Myeloablative	No	2	No	Alive	Alive	>+638
54	61	FISH del7q	sAML	No	CR1	4% mb	**46, xy,+1, der (1,7) (q10;p10)**	**Abn. signal pattern 7q, +21q**	R 8/8 PBSC	TBI/Myeloablative	No	4	No	+78	I, G, OF	+78
58	65	del20q	sAML	No	CR2	**4% abn. mb**	**46, xy, del (20) (q11.2q13.3)**	/	U 8/8 BM	NonTBI/Myeloablative	No	0	Yes	+385	G, OF	+385

Pt: patient; Dg: Diagnosis; Rem: status remission status; FISH: fluorescence *in situ* hybridization; aGVHD: acute graft-*vs*.-host disease; cGVHD: chronic graft-*vs*.-host disease; TRM: treatment related mortality; OS: overall survival; M: male; F: female; AML: acute myeloid leukemia; sAML: secondary acute myeloid leukemia; Auto SCT: autologous stem cell transplant; CR1: first complete remission; CR2: second complete remission; abn: abnormal; mb: myeloblasts; ck: complex karyotype; R: related; U: unrelated; PBSC: peripheral blood stem cells; BM: bone marrow; CB: cord blood; TBI: total body irradiation; non-TBI: non total body irradiation; L: leukemia; G: GVHD; I: infection; H: hemorrhage; OF: organ failure.

There were a total of 32 (54.2%) deaths, of which 15 were in the MRD-positive group (eight in CR1 and seven in CR2), and 17 in the MRD-negative group (seven deaths were in CR1 and 10 in CR2). A total of 17 (32.8%) relapses occurred, of which 11 were in the MRD-positive group (five in CR1 and six in CR2) and six in the MRD-negative group (two relapses in CR1 and four in CR2). There were 15 (25.4%) TRM events, of which four were in the MRD-positive group and 11 in the MRD-negative group. The main cause of death was relapse in 17 patients (53.1%), GVHD in six patients (18.7%), five patients died of infection (15.6%), two patients of hemorrhage (6.2%) and two of multiorgan failure (6.2%). There are 27 (45.8%) patients alive.

### 3.1. Overall Survival

**Figure 1:**
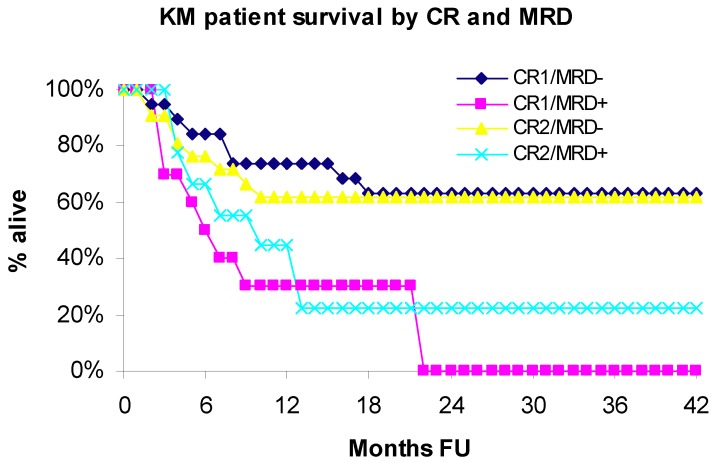
Overall survival by CR and MRD.

Median overall survival (OS) was 21.8 months (range 1.4–125.9 months), with significantly better survival in the group without MRD (*p* < 0.05). Overall survival was significantly lower in MRD-positive compared to MRD-negative patients (*p* = 0.0032). The rate of mortality was 2.97 times greater among patients with MRD *vs*. those without MRD (HR = 2.97; 95% CI: 1.44–6.12). The actuarial OS at 3-years was 48.6% in the entire study group, 17.5% in MRD-positive group *vs*. 62.3% in MRD-negative group. For MRD-negative group, the 5 year survival was 54.5% and 10 year survival was 46.7%. Overall survival curves estimated using the Kaplan Meier (KM) method for patients in first (CR1) or second (CR2) complete remission, with (MRD+) or without (MRD−) evidence of minimal residual disease (MRD) are shown in Figure 1.

In the CR1 subset, the rate of mortality was 3.52 times greater among patients with MRD *vs*. those without MRD (HR = 3.52; 95% CI: 1.25–9.94; *p* = 0.0176). In the CR2 subset, the rate of mortality was 2.4 times greater among patients with MRD *vs*. those without MRD (HR = 2.38; 95% CI: 0.86–6.60; *p* = 0.0943). The last patient in the CR1/MRD+ group was censored at 21.8 months follow-up time. Hence, KM survival estimates beyond 22 months are undefined and not reported. The estimate for OS in 20-months in the CR1/CR2 subsets was: CR1/MRD+ 30% *vs*.CR1/MRD− 63.2% and CR2/MRD+ 22.2% *vs*. CR2/MRD− 61.9%.

### 3.2. Leukemia Free Survival

**Figure 2:**
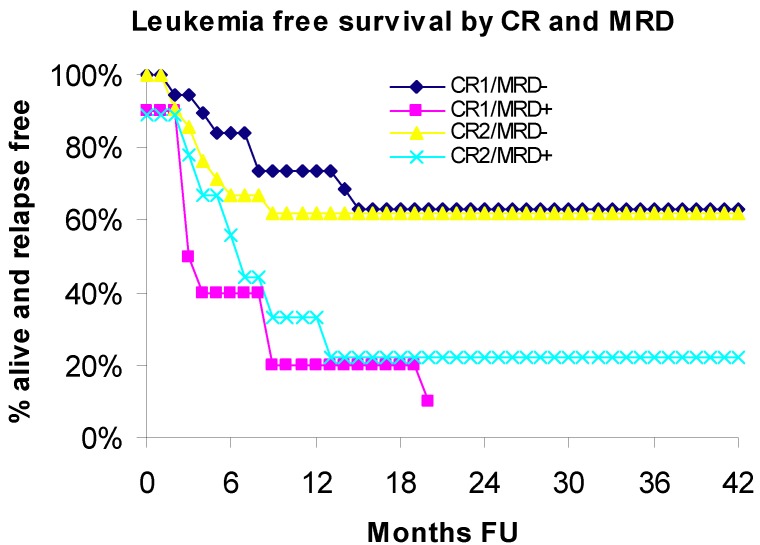
Leukemia free survival by CR and MRD.

Leukemia free survival (LFS) was significantly lower in MRD-positive patients compared to MRD-negative patients (*p* = 0.0006). The rate of relapse/mortality was 3.49 times greater among patients with MRD *vs*. those without MRD (HR = 3.49; 95% CI: 1.71–7.11). The estimate for LFS in 3-years was 47.2% in the entire study group, 15.8% in MRD-positive group *vs*. 62.4% in MRD-negative group. Leukemia-free survival curves estimated using the Kaplan Meier (KM) method for patients in CR1 or CR2, with (MRD+) or without (MRD−) evidence of minimal residual disease (MRD) are shown in Figure 2.

In the CR1 subset, the rate of relapse/mortality was 4.52 times greater among MRD-positive patients compared to MRD-negative patients (HR = 4.52; 95% CI: 1.65–12.36; *p* = 0.0033). In the CR2 subset, the rate of relapse/mortality was 2.45 times greater among MRD-positive patients compared to MRD-negative patients (HR = 2.4; 95% CI: 0.88–6.80; *p* = 0.0845). The last patient in the CR1/MRD+ group was censored at 21.8 months follow-up time. Hence, KM survival estimates beyond 22 months are undefined and not reported. The estimate for LFS in 20-months in the CR1/CR2 subsets was: CR1/MRD+ 10% *vs*. CR1/MRD− 63.2% and CR2/MRD+ 22.2% *vs*. CR2/MRD− 61.9%.

### 3.3. Relapse

**Figure 3:**
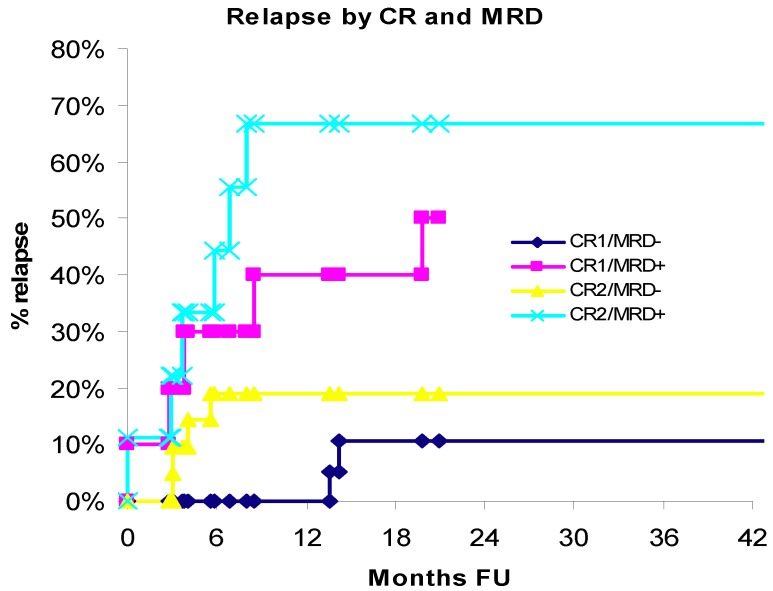
Relapse by CR and MRD.

The likelihood of relapse was significantly greater in patients who were MRD-positive at the time of transplant compared to MRD-negative (HR = 5.26; 95% CI: 1.98–13.97; *p* = 0.001). In the CR1 subset, the rate of relapse was 6.36 times greater among patients with MRD *vs*. those without MRD (HR = 6.36; 95% CI: 1.33–30.34; *p* = 0.020). In the CR2 subset, the rate of relapse was 4.99 times greater among patients with MRD *vs*. those without MRD (HR = 4.99; 95% CI: 1.34–18.53; *p* = 0.016). In the CR1/MRD− subset, the patients remained relapse free for 13.5 months *vs*. CR2/MRD− subset was patients remained relapse free for 4.8 months. In the MRD-positive group 11 (57.9%) patients relapsed at a median time of 3.7 months and eight (42.1%) patients remained relapse free for 10.8 months (range: 2.6–49.7) (*p* = 0.0007); in the MRD-negative group, six (15.0%) patients relapsed at a median time of 4.8 months and 34 (85.0%) patients remained relapse free for 34.4 months (range: 1.4–125.9). Cumulative incidence of relapse for patients in CR1 or CR2 complete remission, with (MRD+) or without (MRD−) evidence of minimal residual disease (MRD) is shown in Figure 3.

The last patient in the CR1/MRD+ group was censored at 21.8 months follow-up time. Hence, KM survival estimates beyond 22 months are undefined and not reported. The estimate for relapse in 20-months in the CR1/CR2 subsets was: CR1/MRD+ 50% *vs*. CR1/MRD− 10.5% and CR2/MRD+ 66.7% *vs*. CR2/MRD− 19%.

### 3.4. Treatment-Related Mortality

**Figure 4:**
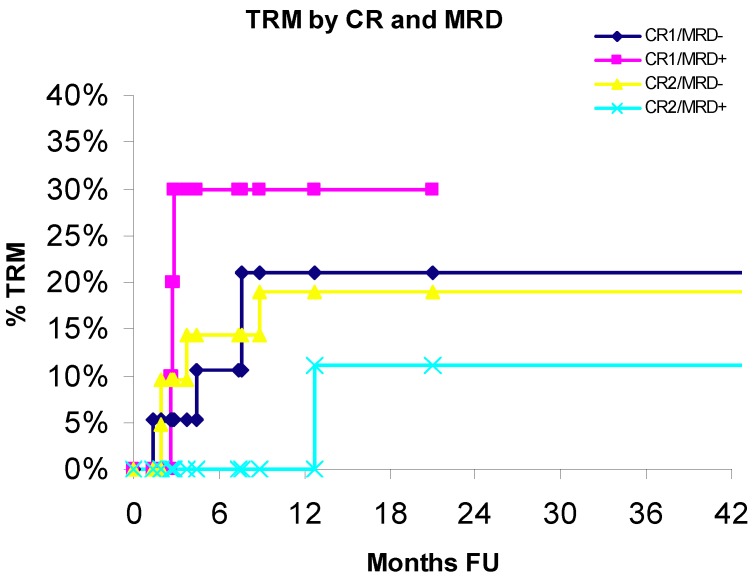
Transplantation related mortality by CR and MRD.

Cumulative incidence of treatment-related mortality (TRM) for patients in CR1 or CR2, with (MRD+) or without (MRD−) evidence of minimal residual disease (MRD) is shown in Figure 4.

TRM incidence did not significantly differ by group (CR1: *p* = 0.552; CR2: *p* = 0.335). The estimate for TRM in 20-months in the CR1/CR2 subsets was: CR1/MRD+ 30% *vs*. CR1/MRD− 21% and CR2/MRD+ 11.1% *vs*. CR2/MRD− 19%.

We compared the risk factors between MRD-positive and MRD-negative subsets: CR1/2, type and source of transplant, HLA matching, initial cytogenetics, previous disease/prior HSCT, conditioning protocol, conditioning intensity and age, as shown in Table 3.

**Table 3:** Comparisons of risk factors between groups.

	**MRD-negative**		**MRD-positive**		
	**(n = 40)**	**(67.8%)**	**(n = 19)**	**(32.2%)**	
	number	%	number	%	***p*-value**
**CR**					
1	19	47.5	10	52.6	0.7126
2	21	52.5	9	47.4	
**Type/source of transplant**					
Related, PBSC	22	55.0	9	47.4	0.0707
Related, BM	0	0.0	3	15.8	
Unrelated, PBSC	5	12.5	1	5.3	
Unrelated, BM	6	15.0	1	5.3	
Unrelated, CB	7	17.5	5	26.3	
**HLA**					
Matched (8/8, 6/6)	32	80.0	14	73.7	0.7282
Mismatched (7/8, 5/6)	3	7.5	4	21.1	
Mismatched (4/6)	5	12.5	1	5.3	
**Initial cytogenetics**					
Favorable	1	2.5	0	0.0	0.2145
Intermediate	24	60.0	9	47.4	
Unfavorable	11	27.5	8	42.1	
Unknown	4	10.0	2	10.5	
**Previous disease/HSCT**					
AML *de novo*	27	67.5	8	42.1	0.1016
Secondary AML	8	20.0	8	42.1	
Prior HSCT	5	12.5	3	15.8	
**Conditioning protocol**					
Non TBI	23	57.5	9	47.4	0.4655
TBI	17	42.5	10	52.6	
**Conditioning intensity**					
Myeleoblative	36	90.0	16	84.2	0.6702
Reduced intensity	4	10.0	3	15.8	
	**Mean**	**SD**	**Mean**	**SD**	
**Age**	44.45	11.8	50.05	10.6	0.0836

Most risk factors were roughly comparable in the two groups. However, groups differed by type/source of transplant (*p* = 0.07); 26.3% of MRD-positive patients were transplanted with unrelated CB compared to 17.5% of MRD-negative patients and were more likely to be HLA mismatched-7/8, 5/6 (21.1% *vs*. 7.5%). In addition, MRD-positive patients tended to be slightly older than MRD-negative patients by roughly five years (*p* = 0.08). It appears that MRD-positive patients tended to be more likely to have unfavorable karyotype of AML at diagnosis/initial cytogenetics (48% *vs*. 30%) and to have secondary AML (43% *vs*. 23%) compared to MRD-negative patients, although the differences did not reach statistical significance.

We compared mortality outcomes in patients with and without MRD after considering the following known and potential risk factors: CR1/2, initial cytogenetics, previous disease/prior SCT, type and source of stem cells, HLA matching, conditioning intensity and conditioning protocol. Age at transplant was also considered and analyzed as a continuous variable. Table 4 shows the univariate analysis of risk factors (HRs with the 95% confidence intervals and *p*-values).

**Table 4:** Univariate Cox regression.

Factor	Unadjusted			
	Hazard Ratio	HRLowerCL	HRUpperCL	*p*-value
**MRD**	2.97	1.44	6.12	**0.0032**
**CR:** 2 *vs*. 1	1.08	0.54	2.17	0.8277
**Initial cytogenetics**				
Unknown *vs*. favorable/intermediate	0.97	0.28	3.35	0.9655
Unfavorable *vs*. favorable/intermediate	1.78	0.85	3.72	0.1238
**Previous disease/prior HSCT**				
Secondary *vs*. *de novo* AML	1.52	0.71	3.22	0.2782
Previous SCT *vs*. *de novo* AML	0.71	0.21	2.42	0.5813
**Age** (per year)	1.03	1.00	1.07	**0.0502**
**Transplant type, source and HLA**				
Type: unrelated *vs*. related	0.49	0.23	1.04	**0.0638**
Source: CB *vs*. PBSC and BM	0.19	0.04	0.79	**0.0225**
HLA: mismatched *vs*. matched	0.27	0.08	0.89	**0.0310**
**Conditioning protocol**				
TBI *vs*. non TBI	0.83	0.41	1.67	0.5981
**Conditioning intensity**				
Reduced intensity *vs*. myeloablative	1.07	0.37	3.08	0.9018

MRD-positive patients had a 2.97 times increased rate of mortality compared to MRD-negative patients (HR = 2.97; 95% CI: 1.44–6.12; *p* = 0.003). Older age was associated with a roughly 3% increase in the rate of mortality per one year increase (HR = 1.03; 95% CI: 1.00–1.07; *p* < 0.050). Patients transplanted with cord blood were at lower risk of adverse outcome. Unfavorable karyotype of AML at diagnosis was associated with a 1.78 times greater rate of mortality compared to intermediate/favorable cytogenetics, although this finding was not significant at *p* < 0.05 level. Secondary AML diagnosis (*vs*. AML *de novo*) was associated with a 1.52 times greater rate of mortality although the difference did not reach statistical significance.

In the final multivariate model only MRD was significant at *p* < 0.05. None of the other factors were significant when controlled for simultaneously in the multivariate model. However, even when the factors found to be significant or marginally significant in the bivariate analysis were forced into the multivariate model, MRD-positive patients still had 3.3 times greater rate of mortality compared to MRD-negative patients. (HR = 3.3; 95% CI: 1.45–7.57; *p* = 0.0044)

## 4. Discussion

In this single institution study we investigated the prognostic value of MRD in adult AML patients undergoing allo-SCT in CR1 and CR2. Although this is a relatively small study, we can clearly see the prognostic impact of minimal residual disease in adult allo-SCT AML patients. Compared to patients who had no detectible residual leukemia, those who were positive for residual disease had significantly increased risk of relapse and death (HR = 3.3; 95% CI: 1.45–7.57; *p* = 0.0044). MRD-positive patients had considerably worse outcome compared to MRD-negative patients, with 3-years LFS of 15.8% *vs*. 62.4% and OS of 17.5% *vs*. 62.3%. Furthermore, relapse rate was significantly higher in MRD-positive patients; 3 years relapse rate in MRD-positive patients was 57.9% *vs*. 15.1% in MRD-negative patients. TRM incidence did not significantly vary by MRD/CR groups. Evidence of MRD correlated with other adverse risk factors in the investigated group. Older age, type/source of transplant, secondary AML and unfavorable karyotype of AML at diagnosis tended to be bivariately predictive for poor outcome. Similar to our study, Walter *et al*. [16] findings suggest that detection of any MRD by flow cytometry at the time of SCT defines a population of patients with AML who are at higher risk for adverse outcome with 4 times greater rate of mortality, with 2-year estimates of overall survival 30.2% and 76.6% for MRD-positive patients and MRD-negative patients, and 2-year estimates of relapse 64.9% and 17.6%, respectively.

Our study showed that for patients in morphologic CR1 and CR2, the presence of minimal residual disease at 1% or greater, as detected by sensitive flow cytometric assays, cytogenetics and FISH, predicts for a significantly increased risk of relapse and reduced OS and LFS. Patients in the CR1 subset were at higher risk of relapse/mortality due to MRD than patients in CR2. There is no doubt that a validated flow cytometry assay is an excellent tool for a rapid MRD evaluation, targeting patients across all genetic subgroups. Our study showed no statistically significant difference in techniques used to demonstrate MRD in predicting adverse outcome. Abnormal cytogenetics found at the time of morphologic CR has been shown to predict shorter overall survival and a higher relapse rate in the patients with AML in our and other studies [16,17]. The use of both conventional cytogenetics analysis and FISH, may overcome the limitation of lower type of sensitivity compared to flow cytometry, and can be an important methods for evaluation of MRD [18,19]. The previously described methods in detection of MRD in AML patients should be further investigated because each of them has its advantages and disadvantages and need further validation [6,7,8,20,21]. Some studies suggests that MRD-positive patients should be identified early after the first induction therapy and assigned alternative and salvage treatment strategies [22], although other studies prefer MRD monitoring at the end of treatment [23,24]. Perea *et al*. [24] showed that relapses were more common in patients with FC MRD level >0.1% at the end of chemotherapy treatment than in patients with <0.1%; cumulative incidence of relapse was 67% and 21% (*p* = 0.03), respectively. Different cut-off levels has been proposed by different authors, a level of >0.1% in our study and others [16,21,24,25], whereas some other studies suggests the cut-off value of 0.035% [23,26,27]. The cut-off value can give us suggestions how to proceed further with MRD-positive patients [21,28]. However, additional larger and prospective studies with all previously mentioned methods for detection of MRD, including molecular PCR methodology are required to establish a universal cut-off value to define a significant MRD level. Although PCR methodology is the most sensitive in detecting of MRD in AML, still nearly 40% of patients with AML have no cytogenetic or molecular markers suitable for PCR monitoring [9]. The use of multiple approaches simultaneously can increase the number of patients who can be studied and balance the limitations of individual methods.

Presence of MRD in pre allo-SCT AML patients defines a high risk group of patients. Further therapeutic possibilities in these patients, such as post-transplant donor lymphocyte infusions (DLI), alternative high dose conditioning regimens, adjuvant treatments, or other early therapeutic intervention should be considered. Recent reports on the success of DLI are encouraging to prevent/delay relapse in AML, achieving OS of up to 50% in selected patients [29], although other studies suggests that DLI is often associated with high rates of GVHD [30]. Second allograft is an acceptable and promising approach in the patients relapsing >6 months after the first allograft, with OS up to 40% [29,30]. Donor-derived natural killer cells can mediate beneficial graft *vs*. leukemia reactions, without GVHD [30]. Other agents, such as 5-azacytidine have been investigated in ongoing phase II clinical trial evaluation their efficacy to treat MRD based on a decreasing CD34-donor chimerism after allo-SCT [30].

## 5. Conclusions

Our study showed that MRD-positivity in adult AML patients in CR1 and CR2 can predict adverse outcome, with increased overall mortality and relapse, even after controlling for other risk factors. Although these findings should be confirmed in a larger study, they provide the basis for further studies that can allow standardization and simplification of the MRD techniques, which can be used to identify the patients who would need further treatment and proper therapy to be applied.
